# *N* ^1^-(4-Methyl­phen­yl)piperidine-1,4-dicarboxamide

**DOI:** 10.1107/S1600536812047836

**Published:** 2012-11-24

**Authors:** Arun M. Islor, M. R. Jagadeesh, H. M. Suresh Kumar, R. Ananda Kumari, Thomas Gerber, Eric Hosten, Richard Betz

**Affiliations:** a National Institute of Technology-Karnataka, Department of Chemistry, Medicinal Chemistry Laboratory, Surathkal, Mangalore 575 025, India; b GM Institute of Technology, Department of Physics, Davangere 577 006, India; c Siddaganga Institute of Technololgy, Department of Physics, Tumkur 572 103, India; d Sree Siddaganga College for Women, Tumkur 572 103, India; e Nelson Mandela Metropolitan University, Summerstrand Campus, Department of Chemistry, University Way, Summerstrand, PO Box 77000, Port Elizabeth, 6031, South Africa

## Abstract

In the title compound, C_14_H_19_N_3_O_2_, the heterocycle adopts a ^1^ *C*_4_ conformation with the N atom being one of the flap atoms. In the crystal, classical N—H⋯O hydrogen bonds and C—H⋯O contacts connect the mol­ecules into a three-dimensional network.

## Related literature

For the pharmacological importance of piperidine and its derivatives, see: Chen *et al.* (2012 ▶); Boja *et al.* (2011 ▶); Jakubowska *et al.* (2012 ▶). For puckering analysis of six-membered rings, see: Cremer & Pople (1975 ▶); Boeyens (1978 ▶). For graph-set analysis of hydrogen bonds, see: Etter *et al.* (1990 ▶); Bernstein *et al.* (1995 ▶).
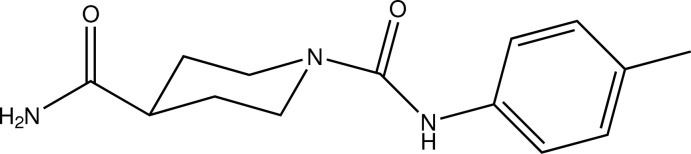


## Experimental

### 

#### Crystal data

C_14_H_19_N_3_O_2_
*M* *_r_* = 261.32
Monoclinic, 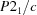

*a* = 5.0102 (1) Å
*b* = 28.6642 (7) Å
*c* = 10.1131 (2) Å
β = 103.113 (1)°
*V* = 1414.50 (5) Å^3^
*Z* = 4
Mo *K*α radiation
μ = 0.08 mm^−1^
*T* = 200 K
0.42 × 0.25 × 0.11 mm

#### Data collection

Bruker APEXII CCD diffractometer
Absorption correction: multi-scan (*SADABS*; Bruker, 2008 ▶) *T*_min_ = 0.966, *T*_max_ = 0.991
27256 measured reflections
3556 independent reflections
3014 reflections with *I* > 2σ(*I*)
*R*_int_ = 0.020

#### Refinement

*R*[*F* ^2^ > 2σ(*F* ^2^)] = 0.044
*wR*(*F* ^2^) = 0.124
*S* = 1.06
3556 reflections
185 parameters
H atoms treated by a mixture of independent and constrained refinement
Δρ_max_ = 0.31 e Å^−3^
Δρ_min_ = −0.19 e Å^−3^

### 

Data collection: *APEX2* (Bruker, 2010 ▶); cell refinement: *SAINT* (Bruker, 2010 ▶); data reduction: *SAINT*; program(s) used to solve structure: *SHELXS97* (Sheldrick, 2008 ▶); program(s) used to refine structure: *SHELXL97* (Sheldrick, 2008 ▶); molecular graphics: *ORTEP-3* (Farrugia, 2012 ▶) and *Mercury* (Macrae *et al.*, 2008 ▶); software used to prepare material for publication: *SHELXL97* and *PLATON* (Spek, 2009 ▶).

## Supplementary Material

Crystal structure: contains datablock(s) I, global. DOI: 10.1107/S1600536812047836/bg2489sup1.cif

Supplementary material file. DOI: 10.1107/S1600536812047836/bg2489Isup2.cdx

Structure factors: contains datablock(s) I. DOI: 10.1107/S1600536812047836/bg2489Isup3.hkl

Supplementary material file. DOI: 10.1107/S1600536812047836/bg2489Isup4.cml

Additional supplementary materials:  crystallographic information; 3D view; checkCIF report

## Figures and Tables

**Table 1:** Hydrogen-bond geometry (Å, °)

*D*—H⋯*A*	*D*—H	H⋯*A*	*D*⋯*A*	*D*—H⋯*A*
N1—H1⋯O1^i^	0.834 (17)	2.128 (17)	2.9481 (13)	167.9 (15)
N3—H3*A*⋯O2^ii^	0.886 (18)	2.071 (18)	2.9451 (14)	168.9 (15)
N3—H3*B*⋯O2^iii^	0.890 (17)	2.034 (17)	2.8875 (13)	160.3 (15)
C3—H3*C*⋯O1^i^	0.99	2.41	3.2987 (17)	149

Symmetry codes: (i) 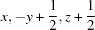; (ii) 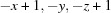; (iii) 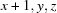.

**Fig. 1.**
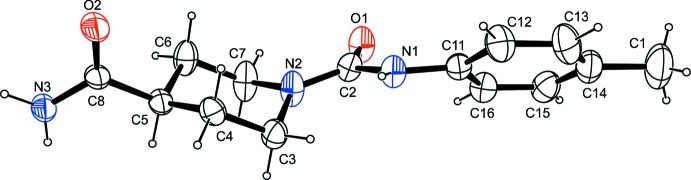
The molecular structure of the title compound, with atom labels and anisotropic displacement ellipsoids (drawn at 50% probability level).

**Fig. 2.**
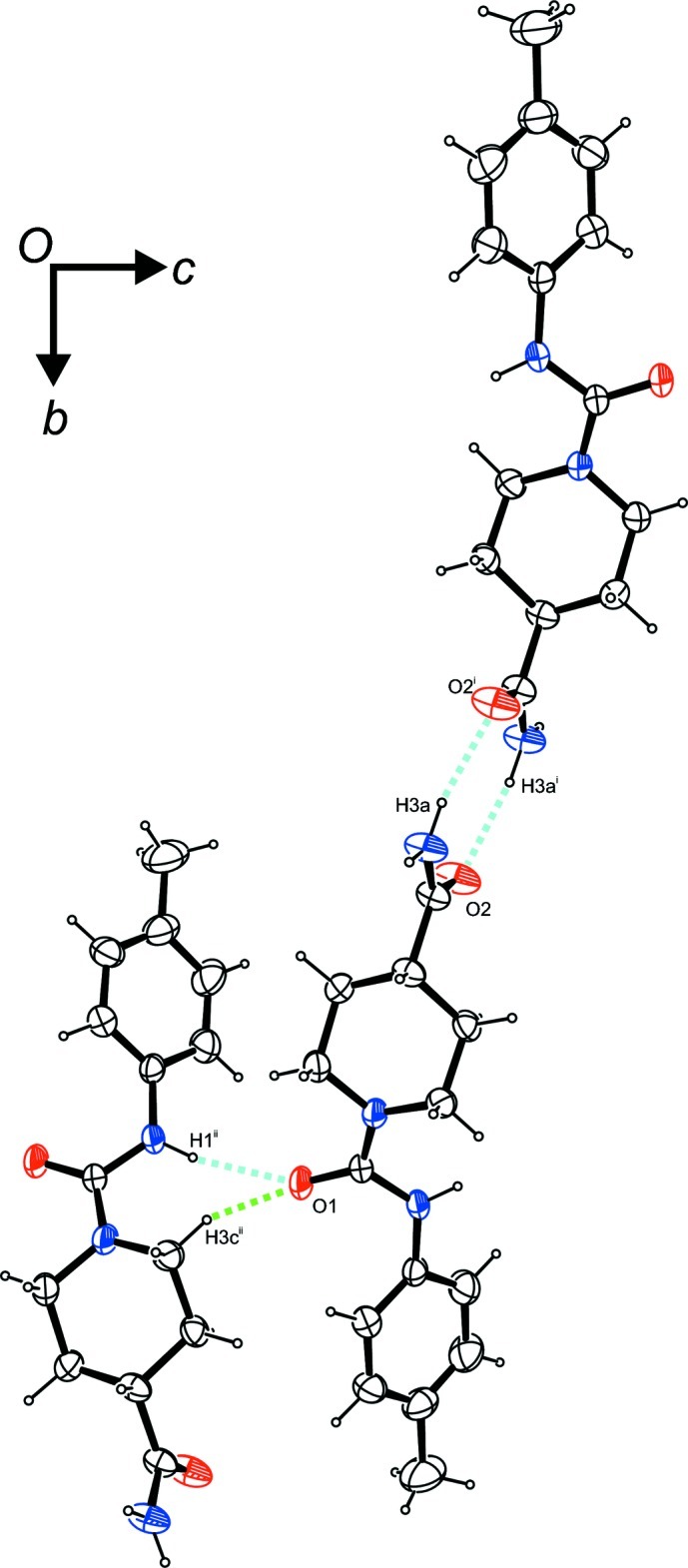
Intermolecular contacts, viewed along [-1 0 0]. For reasons of clarity, only a selection of contacts is shown. Blue dashed lines illustrate classical hydrogen bonds of the N–H···O type, green dashed lines depict C–H···O contacts. Symmetry operators: i -x + 1, -y, -z + 1; iix, -y + 1/2, z - 1/2.

## supplementary crystallographic information

### Comment

Piperidine and its derivatives are ubiquitous building blocks in the synthesis of pharmaceuticals and fine chemicals (Chen *et al.*, 2012; Boja *et al.*, 2011, Jakubowska *et al.*, 2012). Members of this family have found a wide range of applications in pharmacology and are used as antidepressants (*e.g.* Paroxetine) and analgesics (*e.g.* meperidine hydrochloride) or to control attention-deficit hyperactivity disorder (*e.g.* Methylphenidate). In view of the biological importance, the title compound was synthesized to study its crystal structure.

According to a puckering analysis (Cremer & Pople, 1975; Boeyens, 1978), the piperidine ring adopts a ^1^*C*_4_ conformation with the the nitrogen atom as well as the carbon atom in *para* position to it acting as the flap atoms (^N2^*C*_C5_). The primary amide group occupies an equatorial position. Due to amide-type resonance, the intracyclic nitrogen atom is present in an almost planar environment, the least-squares plane defined by the urea moiety (N2–C2–O1–N1) featuring the carbon atom as the one atom deviating most from this plane by 0.010 (1) Å (r.m.s. of all fitted atoms = 0.0057 Å). The least-squares planes through the atoms of the heterocycle and the phenyl groups define an angle of 48.15 (7) °. The planes defined by the non-hydrogen atoms of the amide groups intersect the least-squares plane defined by the intracyclic atoms of the heterocycle at angles of 29.22 (15) ° and 71.8 (2) ° with the smaller angle found for the secondary amide group (Fig. 1).

In the crystal, non-classical C–H..O bonds as well as classical hydrogen bonds of the N–H···O type coexist. The former ones take part between one of the intracyclic methylene groups directly bonded to the nitrogen atom of the piperidine moiety and the oxygen atom of the secondary amide group (which also acts as acceptor for one set of N–H···O hydrogen bonds). The hydrogen atoms of the primary amide group, in turn, link the oxygen atom of its own functional group in neighbouring molecules as acceptor. Metrical parameters as well as information about the symmetry of these contacts are summarized in Table 1. In total, these contacts connect the molecules to a three-dimensional network. According to a graph-set analysis (Etter *et al.*, 1990; Bernstein *et al.*, 1995), the descriptor for the C–H···O contacts is *C*^1^_1_(5) on the unitary level while the descriptor found for the hydrogen bonds fostered by the secondary amide group necessitates a *C*^1^_1_(4) on the same level. The description of the hydrogen bonding pattern created by the primary amide group is best achieved by a binary descriptor of *R*^2^_4_(8). The shortest intercentroid distance between two aromatic systems corersponds to a [100] translation (Fig. 2).

### Experimental

Piperidine-4-carboxamide (10.0 g, 0.078 mol) was dissolved in THF (200 ml). To this triethylamine (23.27 g, 0.23 mol) was added, followed by 1-isocyanato-4-methylbenzene (11.31 g, 0.085 mol). The reaction mixture was stirred at room temperature for 12 h. Completion of the reaction was monitored by TLC. The precipitated solid was filtered, washed with THF and dried under vacuum to get the desired product. The resulting solid was recrystallized from ethanol, yield: 18.5 g (90.77%).

### Refinement

Carbon-bound H atoms were placed in calculated positions (C–H 0.95 Å for aromatic carbon atoms, C–H 0.99 Å for methylene groups and C–H 1.00 Å for methine groups) and were included in the refinement in the riding model approximation, with *U*(H) set to 1.2*U*_eq_(C). The H atoms of the methyl groups were allowed to rotate with a fixed angle around the C–C bond to best fit the experimental electron density (HFIX 137 in the *SHELX* program suite (Sheldrick, 2008)), with *U*(H) set to 1.5*U*_eq_(C). All nitrogen-bound H atoms were located on a difference Fourier map and refined freely.

### Crystal data

**Table:**

C_14_H_19_N_3_O_2_	*F*(000) = 560
*M**_r_* = 261.32	*D*_x_ = 1.227 Mg m^−^^3^
Monoclinic, *P*2_1_/*c*	Melting point = 523–521 K
Hall symbol: -P 2ybc	Mo *K*α radiation, λ = 0.71073 Å
*a* = 5.0102 (1) Å	Cell parameters from 9992 reflections
*b* = 28.6642 (7) Å	θ = 2.2–28.3°
*c* = 10.1131 (2) Å	µ = 0.08 mm^−^^1^
β = 103.113 (1)°	*T* = 200 K
*V* = 1414.50 (5) Å^3^	Platelet, colourless
*Z* = 4	0.42 × 0.25 × 0.11 mm

### Data collection

**Table:**

Bruker APEXII CCD diffractometer	3556 independent reflections
Radiation source: fine-focus sealed tube	3014 reflections with *I* > 2σ(*I*)
Graphite monochromator	*R*_int_ = 0.020
φ and ω scans	θ_max_ = 28.4°, θ_min_ = 2.2°
Absorption correction: multi-scan (*SADABS*; Bruker, 2008)	*h* = −6→6
*T*_min_ = 0.966, *T*_max_ = 0.991	*k* = −38→38
27256 measured reflections	*l* = −13→13

### Refinement

**Table:**

Refinement on *F*^2^	Primary atom site location: structure-invariant direct methods
Least-squares matrix: full	Secondary atom site location: difference Fourier map
*R*[*F*^2^ > 2σ(*F*^2^)] = 0.044	Hydrogen site location: inferred from neighbouring sites
*wR*(*F*^2^) = 0.124	H atoms treated by a mixture of independent and constrained refinement
*S* = 1.06	*w* = 1/[σ^2^(*F*_o_^2^) + (0.0614*P*)^2^ + 0.3543*P*] where *P* = (*F*_o_^2^ + 2*F*_c_^2^)/3
3556 reflections	(Δ/σ)_max_ < 0.001
185 parameters	Δρ_max_ = 0.31 e Å^−^^3^
0 restraints	Δρ_min_ = −0.19 e Å^−^^3^

### Fractional atomic coordinates and isotropic or equivalent isotropic displacement parameters (Å^2^)

**Table:**

	*x*	*y*	*z*	*U*_iso_*/*U*_eq_	
O1	0.1615 (2)	0.25725 (3)	0.13744 (9)	0.0434 (2)	
O2	0.31030 (17)	0.05523 (3)	0.44661 (12)	0.0459 (3)	
N1	0.1515 (2)	0.27199 (3)	0.35689 (10)	0.0330 (2)	
H1	0.155 (3)	0.2596 (5)	0.4318 (17)	0.042 (4)*	
N2	0.3917 (3)	0.20893 (4)	0.30192 (10)	0.0394 (3)	
N3	0.7377 (2)	0.03696 (4)	0.43233 (13)	0.0386 (3)	
H3A	0.710 (3)	0.0081 (6)	0.4578 (17)	0.049 (4)*	
H3B	0.898 (3)	0.0466 (6)	0.4190 (16)	0.045 (4)*	
C1	−0.2229 (5)	0.46134 (6)	0.3273 (2)	0.0767 (6)	
H1A	−0.0793	0.4818	0.3082	0.115*	
H1B	−0.2613	0.4698	0.4150	0.115*	
H1C	−0.3898	0.4649	0.2556	0.115*	
C2	0.2337 (3)	0.24678 (4)	0.25859 (11)	0.0310 (2)	
C3	0.5469 (3)	0.20076 (4)	0.44066 (12)	0.0368 (3)	
H3C	0.4867	0.2229	0.5031	0.044*	
H3D	0.7444	0.2060	0.4461	0.044*	
C4	0.5025 (2)	0.15119 (4)	0.48297 (12)	0.0321 (2)	
H4A	0.3070	0.1467	0.4839	0.039*	
H4B	0.6129	0.1456	0.5760	0.039*	
C5	0.5848 (2)	0.11644 (4)	0.38535 (12)	0.0301 (2)	
H5	0.7851	0.1202	0.3899	0.036*	
C6	0.4276 (3)	0.12705 (4)	0.24063 (13)	0.0409 (3)	
H6A	0.4911	0.1060	0.1765	0.049*	
H6B	0.2297	0.1213	0.2327	0.049*	
C7	0.4714 (4)	0.17763 (4)	0.20382 (14)	0.0472 (4)	
H7A	0.6666	0.1827	0.2034	0.057*	
H7B	0.3604	0.1845	0.1117	0.057*	
C8	0.5337 (2)	0.06691 (4)	0.42456 (12)	0.0305 (2)	
C11	0.0476 (2)	0.31796 (4)	0.34039 (11)	0.0302 (2)	
C12	−0.1339 (3)	0.33220 (5)	0.41605 (15)	0.0455 (3)	
H12	−0.2011	0.3104	0.4713	0.055*	
C13	−0.2187 (4)	0.37835 (6)	0.41185 (17)	0.0544 (4)	
H13	−0.3429	0.3877	0.4653	0.065*	
C14	−0.1276 (3)	0.41117 (5)	0.33202 (15)	0.0482 (3)	
C15	0.0491 (3)	0.39607 (5)	0.25471 (15)	0.0452 (3)	
H15	0.1115	0.4177	0.1972	0.054*	
C16	0.1383 (3)	0.35025 (4)	0.25839 (13)	0.0383 (3)	
H16	0.2618	0.3409	0.2046	0.046*	

### Atomic displacement parameters (Å^2^)

**Table:**

	*U*^11^	*U*^22^	*U*^33^	*U*^12^	*U*^13^	*U*^23^
O1	0.0715 (7)	0.0391 (5)	0.0191 (4)	0.0123 (4)	0.0091 (4)	0.0027 (3)
O2	0.0244 (4)	0.0361 (5)	0.0818 (7)	0.0014 (3)	0.0219 (4)	0.0156 (4)
N1	0.0484 (6)	0.0318 (5)	0.0201 (5)	0.0058 (4)	0.0106 (4)	0.0038 (4)
N2	0.0650 (7)	0.0307 (5)	0.0221 (5)	0.0115 (5)	0.0094 (5)	0.0012 (4)
N3	0.0236 (5)	0.0294 (5)	0.0654 (8)	0.0017 (4)	0.0159 (5)	0.0104 (5)
C1	0.1017 (15)	0.0434 (9)	0.0764 (13)	0.0245 (9)	0.0024 (11)	−0.0068 (8)
C2	0.0435 (6)	0.0291 (5)	0.0209 (5)	−0.0002 (4)	0.0083 (4)	0.0012 (4)
C3	0.0458 (7)	0.0307 (6)	0.0301 (6)	0.0028 (5)	0.0011 (5)	0.0018 (4)
C4	0.0347 (6)	0.0338 (6)	0.0265 (6)	0.0043 (4)	0.0042 (4)	0.0054 (4)
C5	0.0241 (5)	0.0282 (5)	0.0410 (6)	0.0009 (4)	0.0135 (4)	0.0063 (4)
C6	0.0620 (8)	0.0311 (6)	0.0324 (6)	0.0079 (5)	0.0162 (6)	−0.0017 (5)
C7	0.0824 (10)	0.0338 (6)	0.0312 (6)	0.0140 (6)	0.0253 (7)	0.0051 (5)
C8	0.0223 (5)	0.0293 (5)	0.0412 (6)	−0.0004 (4)	0.0096 (4)	0.0059 (4)
C11	0.0347 (5)	0.0326 (5)	0.0219 (5)	0.0022 (4)	0.0033 (4)	−0.0010 (4)
C12	0.0540 (8)	0.0451 (7)	0.0429 (7)	0.0076 (6)	0.0224 (6)	0.0027 (6)
C13	0.0629 (9)	0.0538 (9)	0.0502 (9)	0.0195 (7)	0.0205 (7)	−0.0047 (7)
C14	0.0566 (8)	0.0371 (7)	0.0438 (8)	0.0100 (6)	−0.0032 (6)	−0.0054 (6)
C15	0.0570 (8)	0.0345 (6)	0.0403 (7)	−0.0013 (6)	0.0035 (6)	0.0052 (5)
C16	0.0460 (7)	0.0368 (6)	0.0330 (6)	0.0014 (5)	0.0113 (5)	0.0029 (5)

### Geometric parameters (Å, º)

**Table:**

O1—C2	1.2333 (14)	C4—H4B	0.9900
O2—C8	1.2360 (13)	C5—C8	1.5114 (15)
N1—C2	1.3659 (15)	C5—C6	1.5270 (17)
N1—C11	1.4127 (15)	C5—H5	1.0000
N1—H1	0.834 (17)	C6—C7	1.5249 (18)
N2—C2	1.3560 (15)	C6—H6A	0.9900
N2—C7	1.4588 (16)	C6—H6B	0.9900
N2—C3	1.4605 (15)	C7—H7A	0.9900
N3—C8	1.3235 (14)	C7—H7B	0.9900
N3—H3A	0.886 (18)	C11—C12	1.3763 (17)
N3—H3B	0.890 (17)	C11—C16	1.3863 (17)
C1—C14	1.513 (2)	C12—C13	1.387 (2)
C1—H1A	0.9800	C12—H12	0.9500
C1—H1B	0.9800	C13—C14	1.383 (2)
C1—H1C	0.9800	C13—H13	0.9500
C3—C4	1.5151 (16)	C14—C15	1.377 (2)
C3—H3C	0.9900	C15—C16	1.3852 (18)
C3—H3D	0.9900	C15—H15	0.9500
C4—C5	1.5238 (16)	C16—H16	0.9500
C4—H4A	0.9900		
			
C2—N1—C11	124.85 (10)	C6—C5—H5	108.5
C2—N1—H1	119.3 (11)	C7—C6—C5	110.61 (11)
C11—N1—H1	115.8 (11)	C7—C6—H6A	109.5
C2—N2—C7	120.14 (10)	C5—C6—H6A	109.5
C2—N2—C3	125.68 (10)	C7—C6—H6B	109.5
C7—N2—C3	112.74 (10)	C5—C6—H6B	109.5
C8—N3—H3A	117.0 (11)	H6A—C6—H6B	108.1
C8—N3—H3B	120.2 (10)	N2—C7—C6	110.00 (10)
H3A—N3—H3B	122.7 (15)	N2—C7—H7A	109.7
C14—C1—H1A	109.5	C6—C7—H7A	109.7
C14—C1—H1B	109.5	N2—C7—H7B	109.7
H1A—C1—H1B	109.5	C6—C7—H7B	109.7
C14—C1—H1C	109.5	H7A—C7—H7B	108.2
H1A—C1—H1C	109.5	O2—C8—N3	122.17 (11)
H1B—C1—H1C	109.5	O2—C8—C5	121.06 (10)
O1—C2—N2	122.28 (11)	N3—C8—C5	116.76 (10)
O1—C2—N1	121.69 (11)	C12—C11—C16	118.82 (11)
N2—C2—N1	116.01 (10)	C12—C11—N1	118.89 (11)
N2—C3—C4	109.95 (10)	C16—C11—N1	122.10 (11)
N2—C3—H3C	109.7	C11—C12—C13	120.15 (13)
C4—C3—H3C	109.7	C11—C12—H12	119.9
N2—C3—H3D	109.7	C13—C12—H12	119.9
C4—C3—H3D	109.7	C14—C13—C12	121.80 (14)
H3C—C3—H3D	108.2	C14—C13—H13	119.1
C3—C4—C5	110.57 (10)	C12—C13—H13	119.1
C3—C4—H4A	109.5	C15—C14—C13	117.23 (13)
C5—C4—H4A	109.5	C15—C14—C1	121.33 (16)
C3—C4—H4B	109.5	C13—C14—C1	121.42 (16)
C5—C4—H4B	109.5	C14—C15—C16	121.86 (13)
H4A—C4—H4B	108.1	C14—C15—H15	119.1
C8—C5—C4	110.93 (9)	C16—C15—H15	119.1
C8—C5—C6	110.94 (10)	C15—C16—C11	120.11 (12)
C4—C5—C6	109.52 (9)	C15—C16—H16	119.9
C8—C5—H5	108.5	C11—C16—H16	119.9
C4—C5—H5	108.5		
			
C7—N2—C2—O1	−3.0 (2)	C4—C5—C8—O2	50.08 (16)
C3—N2—C2—O1	162.27 (13)	C6—C5—C8—O2	−71.88 (15)
C7—N2—C2—N1	175.02 (12)	C4—C5—C8—N3	−130.68 (12)
C3—N2—C2—N1	−19.70 (19)	C6—C5—C8—N3	107.35 (13)
C11—N1—C2—O1	−18.65 (19)	C2—N1—C11—C12	151.54 (13)
C11—N1—C2—N2	163.31 (11)	C2—N1—C11—C16	−33.52 (18)
C2—N2—C3—C4	134.00 (13)	C16—C11—C12—C13	−1.4 (2)
C7—N2—C3—C4	−59.78 (15)	N1—C11—C12—C13	173.76 (13)
N2—C3—C4—C5	57.39 (13)	C11—C12—C13—C14	0.5 (2)
C3—C4—C5—C8	−178.42 (9)	C12—C13—C14—C15	0.9 (2)
C3—C4—C5—C6	−55.63 (13)	C12—C13—C14—C1	179.54 (16)
C8—C5—C6—C7	177.79 (10)	C13—C14—C15—C16	−1.5 (2)
C4—C5—C6—C7	55.01 (13)	C1—C14—C15—C16	179.85 (15)
C2—N2—C7—C6	−133.78 (13)	C14—C15—C16—C11	0.7 (2)
C3—N2—C7—C6	59.15 (16)	C12—C11—C16—C15	0.76 (19)
C5—C6—C7—N2	−56.27 (16)	N1—C11—C16—C15	−174.18 (12)

### Hydrogen-bond geometry (Å, º)

**Table:**

*D*—H···*A*	*D*—H	H···*A*	*D*···*A*	*D*—H···*A*
N1—H1···O1^i^	0.834 (17)	2.128 (17)	2.9481 (13)	167.9 (15)
N3—H3*A*···O2^ii^	0.886 (18)	2.071 (18)	2.9451 (14)	168.9 (15)
N3—H3*B*···O2^iii^	0.890 (17)	2.034 (17)	2.8875 (13)	160.3 (15)
C3—H3*C*···O1^i^	0.99	2.41	3.2987 (17)	149

Symmetry codes: (i) *x*, −*y*+1/2, *z*+1/2; (ii) −*x*+1, −*y*, −*z*+1; (iii) *x*+1, *y*, *z*.
